# Etiologies des œdèmes papillaires en milieu hospitalier camerounais

**DOI:** 10.11604/pamj.2023.45.66.36676

**Published:** 2023-05-29

**Authors:** Godefroy Koki, Hassan Aboubakar, William Nzokou Mukam, Prisca Nyamsi Biangoup, Patrick Tépéresna, Emilienne Epée, Assumpta Lucienne Bella

**Affiliations:** 1 Faculté de Médecine et des Sciences Biomédicales, Université de Yaoundé I, Yaoundé, Cameroun,; 2 Hôpital d´Instruction, d´Application et de Références des Armées de Yaoundé, Yaoundé, Cameroun

**Keywords:** Œdème papillaire, neuropathie optique inflammatoire, étiologies, Yaoundé, Cameroun, Papilledema, inflammatory optic neuropathy, etiologies, Yaoundé-Cameroon

## Abstract

**Introduction:**

l´œdème papillaire (OP) est le gonflement de la tête du nerf optique, signe d´appel majeur de nombreuses pathologies locales, locorégionales ou systémiques pouvant mettre en jeu le pronostic visuel ou vital. Il constitue une urgence diagnostique et thérapeutique présente dans nos services, dont il paraissait important et opportun de déterminer les étiologies les plus rencontrées en milieu hospitalier camerounais.

**Méthodes:**

c´est une étude documentaire et descriptive des patients présentant un œdème papillaire, ayant consulté au service d´ophtalmologie de l´Hôpital d´Instruction, d´Application et de Référence des Armées de Yaoundé du 1^er^ octobre 2013 au 31 décembre 2016. Les variables étudiées portaient sur les données épidémiologiques (âge, sexe), cliniques (signes fonctionnels, acuité visuelle, aspect de la papille et signes associés selon la classification de Hoyt et Beesten), les examens complémentaires réalisés (angiographie à la fluorescéine, champ visuel, biologie, radiographie, tomodensitométrie) et le diagnostic étiologique retenu. Le logiciel Epi-info 3.5.3 a permis l´analyse statistique et le Chi-carré utilisé avec p < 5% significatif.

**Résultats:**

l´œdème papillaire était présent chez 26 sur 5023 patients consultés pendant cette période d´étude, soit une fréquence de 0,5%. La moyenne d´âge était de 32,7 ± 10,9 ans avec des extrêmes de 7 et 79 ans, pour 13 femmes et 13 hommes. L´œdème papillaire était bilatéral chez 15 (57,7%) patients et unilatéral chez 11 (42,3%), soit 41 yeux atteints. Les étiologies étaient faites de 11 (42,3%) neuropathies optiques inflammatoires, 5 (19,2%) hypertensions artérielles, 4 (15,4%) occlusions de la veine centrale de la rétine, 3 (11,5%) contusions oculaires, 2 (7,7%) hydrocéphalies et 1 cas (3,9%) de neuro paludisme.

**Conclusion:**

les neuropathies optiques inflammatoires et vasculaires constituaient les étiologies les plus fréquentes des œdèmes papillaires dans notre contexte.

## Abstract (en)

**Introduction:**

papilledema refers to the swelling of the head of the optic nerve, a major sign of many local, locoregional or systemic pathologies that may involve patients’ visual or vital prognosis. This condition represents a diagnostic and therapeutic emergency frequently encountered in our facilities. Therefore, it is deemed and opportune to identify the most common etiologies observed in Cameroonian hospitals.

**Method:**

we conducted a documentary and descriptive study of patients with papilledema presenting to the Ophthalmology Department of the Hospital of Instruction, Application and Reference of the Armed Forces of Yaoundé from 1^st^ October 2013 to 31^st^ December 2016. The variables under investigation included epidemiological data (age, sex), clinical data (functional signs, visual acuity, appearance of the papilla and associated signs according to the Hoyt and Beesten classification), complementary examinations performed (fluorescein angiography, visual field, biology, radiography, CT scan) and the etiological diagnosis. Epi-info 3.5.3 software was used for statistical analysis and the Chi-square test was performed at a 5% significance level (p < 5%).

**Results:**

during the study period, papilledema was found in 26 out of 5023 patients, reflecting a rate of 0.5%. The average age of patients was 32.7± 10.9 years, ranging from 7 to 79 years, for 13 women and 13 men. Papilledema was bilateral in 15 (57.7%) patients and unilateral in 11 (42.3%), or 41 affected eyes. The etiologies were 11 (42,3%) inflammatory optic neuropathies, 5 (19,2%) arterial hypertension, 4 (15,4%) central retinal vein occlusions, 3 (11,5%) eye contusions, 2 (7,7%) hydrocephalus and 1 case (3,9%) of cerebral malaria.

**Conclusion:**

inflammatory and vascular optic neuropathies were the most common etiologies of papilledema in our facilities.

## Introduction

L´œdème papillaire (OP) est un gonflement de la tête du nerf optique, accompagné ou non de l´accumulation de matériel axoplasmique au niveau de la lame criblée par défaut de transport axonal [1]. Son diagnostic positif repose sur l´examen clinique du fond d´œil (FO) et devant un doute, l´angiographie à la fluorescéine est utile [2]. Il peut être pur (sans atteinte primitive de la fibre optique) ou accompagné (avec atteinte primitive de la fibre optique). Ce signe d´appel majeur de nombreuses pathologies locales, locorégionales ou systémiques pouvant mettre en jeu le pronostic visuel et même vital dans notre milieu, constitue une urgence diagnostique et thérapeutique. Le diagnostic étiologique est guidé par un examen clinique minutieux et des examens complémentaires utiles souvent demandés mais difficilement ou tardivement réalisés par les malades dans notre environnement de pauvreté. Le traitement est essentiellement étiologique [3-6]. C´est ainsi qu´il nous a paru important et opportun de déterminer les étiologies des OP fréquemment retrouvés en milieu hospitalier camerounais afin d´aider à la présomption diagnostique.

## Méthodes

**Conception et type d´étude:** il s´agissait d´une étude documentaire et descriptive réalisée dans le service d´ophtalmologie de l´Hôpital d´Instruction, d´Application et de Référence des Armées de Yaoundé (HIARAY) au Cameroun, qui compilait consécutivement les dossiers des patients ayant consulté entre le 1^er^ octobre 2013 et le 31 décembre 2016 afin de déterminer les étiologies des œdèmes papillaires. Les mots clés (neuropathie optique, hypertension artérielle, occlusion veineuse rétinienne, traumatisme du segment postérieur oculaire) adjoints au terme œdème papillaire ont permis à partir de PubMed et de Google Scholar, une recherche bibliographique.

**Collecte des données:** elle a été faite à partir d´une fiche technique sur papier comportant les données sociodémographiques des patients (âge, sexe, profession), l´examen clinique ophtalmologique (signes fonctionnels, acuité visuelle, aspect de la papille et signes associés selon la classification de Hoyt et Beesten), les examens complémentaires réalisés (angiographie à la fluorescéine, champ visuel, biologie, radiographie, tomodensitométrie) et le diagnostic étiologique retenu. La biologie immunitaire comprenant le dosage d´anticorps anti-Aquaporine 4 (AQP4) et MOG (Glycoprotéine Oligodendrocytaire productrice de la Myéline) n´était pas réalisée parce que récente et non encore disponible dans notre contexte [4]. Les examens biologiques étaient faits de sérologie d´Human Immunodeficiency Virus (HIV), numération formule sanguine, vitesse de sédimentation, sérologies (toxoplasmose, oreillons, rubéole, cytomégalovirus, herpès 1 et 2 (TORCH)), glycémie à jeun ou hémoglobine glyquée et treponema pallidum hemagglutinations assay (TPHA) / venereal disease research laboratory (VDRL).

**Critères d´inclusion et d´exclusion:** tous les dossiers des patients camerounais, de tout âge sans distinction de sexe, comportant un diagnostic d´œdème papillaire cliniquement décrit ou confirmé par l´imagerie pendant la période d´étude étaient retenus. Les faux œdèmes papillaires étaient exclus [7].

**Analyse des données:** le logiciel Epi-info version 3.5.3 a permis l´analyse statistique des données disponibles, et le test utilisé était celui du Chi-carré avec p < 5% significatif.

**Financement:** cette étude n´a bénéficié d´aucun apport financier.

**Ethique:** c´est une étude documentaire qui a bénéficié d´une autorisation de recherche et de publication de l´Hôpital d´Instruction, d´Application et de Référence des Armées de Yaoundé (HIARAY). Elle a été faite dans le respect des conventions nationales et internationales [8].

## Résultats

**Caractéristiques sociodémographiques:** au total, 42 patients dont 26 (41 yeux) sur 5023 patients consultés durant la période d´étude présentaient un œdème papillaire, soit une fréquence de 0,5% et 16 étaient exclus (12 pour faux œdèmes papillaires et 4 pour dossiers imprécis et incomplets). On comptait 13 femmes (50%) et 13 hommes (50%) avec un sex-ratio de 1. La moyenne d´âge était de 32,7± 10,9 ans (extrêmes: 7 ans et 79 ans). La tranche d´âge la plus affectée était celle de 11-20 ans avec 26,9%, suivie de celles de 21-30 et 31-40 ans avec 15,4% chacune et enfin celles de 41-50 et 61-70 avec 11,5% chacune. Les élèves/étudiants étaient les plus concernés avec 11 cas (42,3%), puis venaient les acteurs des secteurs public et privé dans 23,1% des cas chacun et enfin les retraités dans 7,7% des cas.

**Figure 1:**
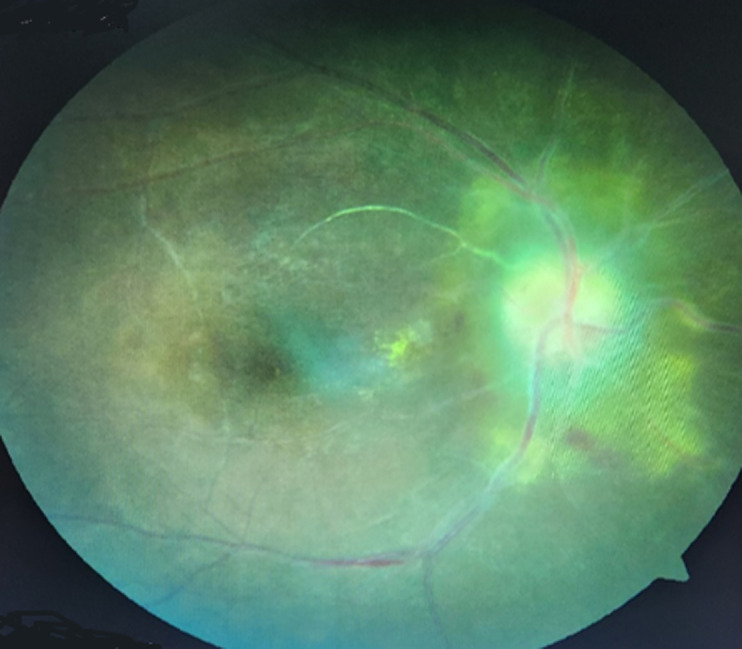
œdème papillaire sur un fond d´œil de patient hypertendu

**Figure 2:**
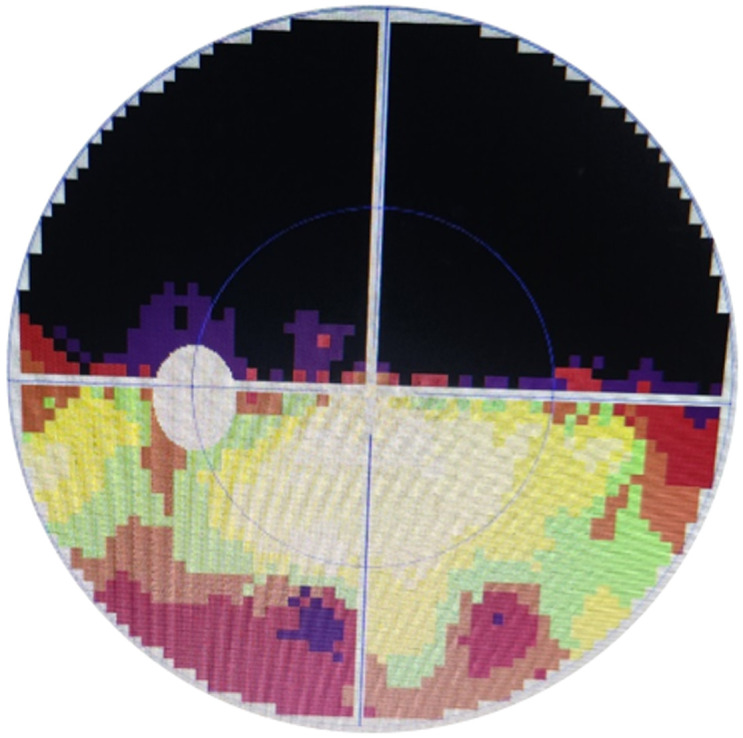
hémianopsie altitudinale supérieure (zone noire) à l´échelle des gris au champ visuel observée au cours d´un œdème papillaire dans un œil gauche

**Caractéristiques cliniques et para cliniques:** le motif de consultation était la baisse d´acuité visuelle (BAV) chez 16 (61,5%) patients, suivi des céphalées chez 6 (23%), de douleurs oculaires chez 4 (15,3%) et d´une amputation du champ visuel chez 1 (3,8%). Les patients référés pour bilan de fond d´œil sur hypertension artérielle étaient 4, soit 15,3%. L´acuité visuelle de loin non corrigée supérieure à 3/10 était observée dans 29 (70,7%) yeux et inférieure à 3/10 dans 12 (29,3%). La cécité était de 17%, soit 7 yeux atteints. Les œdèmes papillaires bilatéraux étaient prédominants, notés chez 15 (57,7%) patients comparés aux unilatéraux chez 11 (42,3%). Selon le Tableau 1, le stade 2 de l´œdème papillaire était le plus fréquent dans 23 (56,1%) yeux. La Figure 1 illustre un cas d´œdème papillaire sur un fond d´œil d´un patient hypertendu. On n´y observe des bords papillaires flous, une disparition des artères dont quelques-unes sont ischémiques (blanches), des vaisseaux engainés et une dilatation veineuse à l´émergence papillaire. Cette description était observée dans 4 yeux. Les examens complémentaires réalisés comprenaient: la biologie (20/26 patients), l´angiographie à la fluorescéine (15/26), le champ visuel à l´exemple de la Figure 2 (13/26), la tomodensitométrie cérébrale (13/26), et la radiographie standard du crâne et de l´orbite (7/26). Les trois premiers étaient systématiquement demandés et les autres étaient fonction de la suspicion diagnostique. Les examens biologiques étaient plus fréquemment réalisés.

**Tableau 1:** répartition des œdèmes papillaires selon les stades cliniques (Hoyt et Beesten)

Stade de l´œdème papillaire (OP)	Effectif	Fréquence (%)
Stade 1: OP débutant	16	39
Stade 2: OP aigu constitué	23	56,1
Stade 3: OP chronique	2	4,9
Stade 4: OP atrophique	0	0,0
**Total**	41	100,0

**Figure 3:**
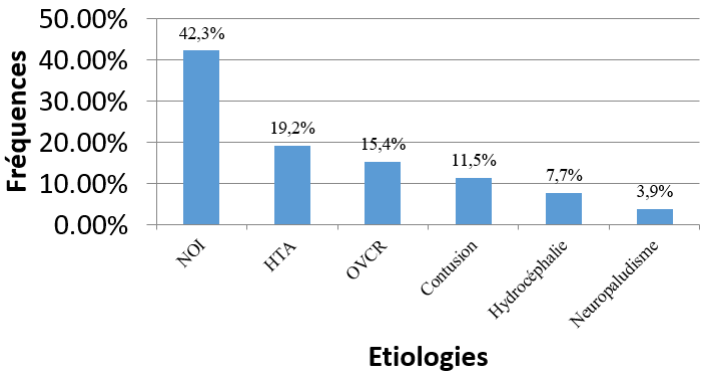
distribution des étiologies

**Etiologies:** les étiologies des OP bilatéraux étaient constituées de 7 patients présentant une neuropathie optique inflammatoire (NOI), 5 pour une hypertension artérielle (HTA), 2 une hydrocéphalie et 1 pour un neuro paludisme. Tandis que celles unilatérales comprenaient 4 patients avec une NOI, 4 une occlusion de veine centrale de la rétine (OVCR) et 3 pour une contusion. La figure 3 montre que les neuropathies optiques inflammatoires étaient les plus fréquentes avec 42,3% de cas soit 11/26 patients. Parmi ceux-ci, 6 étaient porteurs du Virus de l´Immunodéficience Humaine.

## Discussion

La fréquence de 0,5% notée dans ce travail, indique le caractère peu fréquent de cette entité pathologique en milieu hospitalier ophtalmologique camerounais. Elle est observée dans une population relativement jeune en général avec un âge moyen autour de 40 ans, et dans celle en âge scolaire en particulier. Ce résultat est supérieur à celui de Deschamps *et al*. en 2007 qui retrouvaient un âge moyen de 27,6 ans [9] même si Rigi *et al*. affirmaient qu´elle pouvait être rencontrée à tout âge [1]. Aucune dominance de sexe n´a été trouvée, contrairement à la prédominance féminine (61,5%) observée par Tournaire-Marques *et al*. en 2011 à Bordeaux en France [10] et celle masculine évoquée par Atipo-tsiba *et al*. à Brazzaville au Congo en 2016 [11]. Les méthodologies différentes d´une étude à l´autre justifieraient ces variations. Sur le plan clinique ophtalmologique, la baisse d´acuité visuelle notée comme principal motif de consultation était corroborée par les données de la littérature [4,5,7,10]. Il faudrait préciser par ailleurs que devant un OP bilatéral de stase, elle peut être conservée ou peu effondrée selon Rougier [12], tandis qu´elle baissera systématiquement devant un OP d´étiologie inflammatoire ou vasculaire [4,7]. La malvoyance était d´environ 30% et la cécité de 17% selon la CIM-10 [13]. Cette dernière était encore plus élevée dans l´étude de Tournaire-Marques *et al*. avec 39% des cas [10]. Ces résultats sont à prendre avec prudence car les corrections optiques n´étaient pas faites. A l´examen du fond d´œil, les œdèmes papillaires de notre série étaient plus bilatéraux qu´unilatéraux. Ce qui est conforme aux données de la littérature [1,9,11]. Le pronostic fonctionnel et même vital peut être engagé devant un OP surtout bilatéral [14,15]. Parmi les nombreuses classifications [14,16,17] qui aident à quantifier l´OP, celle de Hoyt et Beesten énoncée par Mané et Robert renseigne sur la durée d´évolution tandis que celle de Frisén non prise en compte dans notre étude, présente le degré de sévérité. Elles sont toutes deux indiquées en pratique clinique courante pour évaluer un œdème de stase [14]. Selon le Tableau 1, le stade 2 de Hoyt et Beesten constitué d´hémorragies, d´exsudats cotonneux et durs et d´une excavation papillaire comblée, était le plus fréquent.

Dans ce travail, les examens biologiques étaient les plus réalisés. Pour Tournaire-Marques *et al*. l´angiographie à la fluorescéine fut l´examen le plus pratiqué, suivi de la biologie [10]. Le coût des examens biologiques séparément pris était inférieur à celui de l´angiographie à la fluorescéine dans notre contexte et pourrait expliquer pourquoi les premiers étaient plus réalisés. L´intérêt de l´angiographie étant de distinguer une neuropathie optique ischémique antérieure aiguë (NOIAA) d´une neuropathie optique inflammatoire (NOI) ou d´un œdème papillaire de stase (OPS), par les séquences différentes de remplissage artériel de la tête du nerf optique [7,9]. Deschamps *et al*. ayant constaté que la durée du remplissage de la tête du nerf optique était supérieure de cinq secondes chez 76% des patients avec NOIAA, alors qu´elle était identique pour le groupe témoin, pour les NOI et les OPS [9]. L´analyse du champ visuel au cours des névrites optiques ou NOI montrait des atteintes variables, le scotome central et le déficit altitudinal étant plus fréquents selon Merabtene *et al*. [4]. La figure 2 apporte cette argumentation clinique supplémentaire en faveur de l´inflammation [4,18]. L´imagerie oculaire et ses évolutions à l´aide de la tomographie à cohérence optique (spectral domain, swept source, enhanced depth imaging) ou orbito-cérébrale (tomodensitométrie, angio-TDM, imagerie par résonance magnétique nucléaire ou angio IRMN) sont souvent indispensables pour suspecter ou confirmer une étiologie mais aussi aider au diagnostic différentiel [3,7,17,19-21] même si elle reste d´accès difficile pour de nombreux malades dans notre environnement constituant ainsi une limite de l´étude.

Les OP peuvent être de stase, inflammatoire ou vasculaire [12]. Toutes ces catégories étaient observées chez nos patients à la Figure 3 avec les neuropathies optiques inflammatoires plus fréquentes, suivies des atteintes vasculaires (HTA, OVCR), des contusions oculaires, de l´hydrocéphalie et de neuropaludisme. L´OP au cours du paludisme grave chez l´enfant est un signe clinique non pathognomonique et peu fréquent selon Koki *et al*. [22]. Tournaire-Marques *et al*. retrouvaient plus de neuropathies optiques ischémiques [10] non retrouvées dans notre série. Ceci était justifié très probablement par la prédominance des pathologies infectieuses et de l’environnement sur celles cardiovasculaires. Si le diagnostic de l´œdème papillaire reste clinique en tout lieu, la recherche étiologique nécessite obligatoirement des examens complémentaires [3-5,7,10,12,19-21,2,3]. Selon les contextes, ces derniers pourraient influencer cette fréquence étiologique au vu de l’analyse de notre étude et de celle de Tournaire-Marques *et al*. [10]. Il est donc impératif que les examens demandés, quel que soit le milieu soient tous réalisés afin de permettre une recherche étiologique plus sure et une prise en charge adéquate. Il est à noter par ailleurs que ce travail présente le biais d’incomplétude des informations médicales dans les dossiers, commun à toutes les études documentaires auquel il faudrait ajouter la non disponibilité des examens biologiques immunitaires et celui d’un environnement de pauvreté où la réalisation par le malade de tous les examens complémentaires souvent demandés par le médecin n’est pas toujours effective. Le petit nombre serait aussi justifié par la prise en compte des dossiers uniquement dans le service d´ophtalmologie.

## Conclusion

Les œdèmes papillaires sont certes peu fréquents en milieu hospitalier camerounais mais ils touchent particulièrement le sujet jeune d´âge scolaire. Ils sont une entité pathologique grave car le pronostic fonctionnel et parfois vital peut être mis en jeu. Les étiologies sont dominées par les neuropathies optiques inflammatoires dans notre contexte, certaines sur terrain VIH positif malgré la gratuité instituée du traitement antirétroviral par l´Etat et ses partenaires au développement. Une recherche étiologique rigoureuse devrait donc être faite devant tout œdème de papille uni ou bilatéral de découverte fortuite ou pas afin de permettre une meilleure prise en charge et de préserver la vue et ou la vie du patient.

### Etat des connaissances sur le sujet

- *En ophtalmologie au Cameroun, cette étude vient combler un vide en fournissant des données cliniques récentes de cette pathologie grave;*
- *Dans notre environnement, chaque malade paie pour ses soins; dans ce contexte, l´examen clinique du fond d´œil selon la qualité de sa précision reste le seul gage d´une bonne orientation diagnostique, afin de permettre un début de prise en charge efficace;*
- *Le caractère prédominant des œdèmes papillaires inflammatoires (42,3%) pourrait se justifier dans notre milieu par la prédominance des infections isolées ou communautaires*.

### Contribution de notre étude à la connaissance

- *Au Cameroun, aucune étude sur les œdèmes papillaires n’a été retrouvée sur les 10 dernières années en ophtalmologie;*
- *L’Hypertension Intra crânienne est une cause majeure des œdèmes papillaires;*
- *La prise en charge de l’œdème papillaire est multidisciplinaire et concerne plus souvent les ophtalmologues, les neurologues, les neurochirurgiens, les urgentistes, les réanimateurs et bien d’autres en fonction de la forme clinique*.
